# PCDTBT based solar cells: one year of operation under real-world conditions

**DOI:** 10.1038/srep21632

**Published:** 2016-02-09

**Authors:** Yiwei Zhang, Edward Bovill, James Kingsley, Alastair R. Buckley, Hunan Yi, Ahmed Iraqi, Tao Wang, David G. Lidzey

**Affiliations:** 1 Department of Physics and Astronomy, University of Sheffield, Sheffield, S3 7RH, UK; 2 Ossila Ltd, Kroto Innovation Centre, Broad Lane, Sheffield, S3 7HQ, UK; 3 Department of Chemistry, University of Sheffield, Sheffield S3 7HF, UK; 4 School of Materials Science and Engineering, Wuhan University of Technology, Wuhan, 430070, China

## Abstract

We present measurements of the outdoor stability of PCDTBT:PC_71_BM based bulk heterojunction organic solar cells for over the course of a year. We find that the devices undergo a burn-in process lasting 450 hours followed by a T_S_80 lifetime of up to 6200 hours. We conclude that in the most stable devices, the observed T_S_80 lifetime is limited by thermally-induced stress between the device layers, as well as materials degradation as a result of edge-ingress of water or moisture through the encapsulation.

The past decade has witnessed a steady growth in the efficiency of organic photovoltaics (OPVs), with power conversion efficiencies (PCEs) currently exceeding 10%^1^,^2^,^3^,^4^; a value regarded as a significant step in the so-called 10/10 target for organic photovoltaics (10% efficiency and 10 years lifetime). Whilst efforts to design and prepare high efficiency devices have been a main focus in OPV research, less work has been expended in improving device stability and lifetime. The International Symposium on OPV Stability (ISOS) has now published a consensus on the stability testing protocols recommended for OPV research that provides a set of guidelines for researchers involved in OPV stability studies^5^. Such protocols have since improved the comparability of OPV stability studies conducted by different research groups.

It is clear that the majority of OPV stability studies have been performed under well-controlled laboratory conditions^6^,^7^,^8^,^9^. Although numerous lessons have been learned from in-house and accelerated lifetime studies^10^, systematic studies on the operation and degradation of PSCs during ‘real-world’ outdoor operation is still limited. The most extensive studies on outdoor testing of OPVs over long periods have been performed by the pioneering work of F. C. Krebs and co-workers^11^,^12^,^13^,^14^,^15^,^16^,^17^,^18^,^19^. Here, research has mainly focused on devices based on a blend of the polymer poly(3-hexylthiophene-2,5-diyl) (P3HT) and [6,6]-phenyl-C61-butyric acid methyl ester (PCBM); a material set now considered a prototypical system for OPV research. It has been shown that such devices undergo degradation at different rates when positioned outside in different geographic locations, and that effective device encapsulation is crucial in extending lifetime in outdoor testing^11^,^13^,^14^. The most stable P3HT:PCBM device modules demonstrated a T_S_80 lifetime of over 10,000 hours^14^.

We note that much of the recent progress in OPV research has occurred as a result of the development of new polymeric materials, with many of such materials based on a donor-acceptor (D-A) architecture^20^,^21^,^22^,^23^. One such D-A copolymer used in bulk heterojunction OPVs is the polymer poly[N-9’-heptadecanyl-2,7-carbazole-alt-5,5-(4’,7’-di-2-thienyl-2’,1’,3’-benzothiadiazole)] (PCDTBT)^24^,^25^,^26^. This polymer has been shown to have a high degree of photochemical stability, with recent work under accelerated conditions using a solar simulator suggesting that PCDTBT:PC_71_BM based OPVs could have an operational lifetime of up to 7 years^27^. Notably other studies have extrapolated even longer lifetimes for PCDTBT devices, with operational lifetimes of up to 15 years claimed^28^. Significantly however, such studies have been performed in a well-controlled laboratory; a condition that is very different from the “real-world” in which such devices are eventually expected to operate. Here, the large daily and seasonal temperature fluctuations could eventually result in other degradation mechanisms such as delamination within a multi-layer structure or especially if partial exposure to the atmosphere (particularly water and moisture) occurs during large area processing^29^,^30^,^31^,^32^,^33^,^34^.

To explore the importance of such degradation mechanisms, we have performed an extended outdoor lifetime study of the operation of PCDTBT:PC_71_BM based OPVs over a period of one year (~8800 hours) from 18^th^ September 2014 to 20^th^ September 2015, with devices located in Sheffield, England. To investigate whether the route used to fabricate the OPVs played a significant role in modifying their operational lifetime, the active organic layer was spin-cast from one of three different solvents/solvent blends; namely chlorobenzene (CB), chloroform (CF) and blend of carbon disulfide (CS_2_) and acetone (4:1 volume ratio). Here, the choice of casting solvent has been previously shown to have a marked impact on device efficiency^35^,^36^, however its effect on long-term operation lifetime is unknown.

As we show below, we find that when tested outdoors, PCDTBT:PC_71_BM devices undergo a burn-in stage characterized by an initial rapid loss in efficiency, followed by a period of slower, linear degradation - a result also reported in laboratory testing^37^. We show that during this post-burn-in period, device efficiency fluctuates as a result of seasonal variations in temperature (an effect not observed in laboratory based studies). We find the burn-in period for PSCs made from different solvent systems are comparable, being around 450 hours, with subsequent degradation dynamics (having a T_S_80 lifetime between 5,200 and 6,200 hours) being largely independent of the choice of casting solvent. We speculate that enhanced degradation during the summer months results from a slow breakdown of the device encapsulation or delamination within the device itself caused by repeated thermal cycling. Our study demonstrates therefore that PCDTBT based OPVs have promising stability when tested under real-world conditions, although this currently appears to be limited by the efficiency of our encapsulation materials and techniques with further refinements in testing protocols being required.

## Results and Discussion

**Figure 1:**
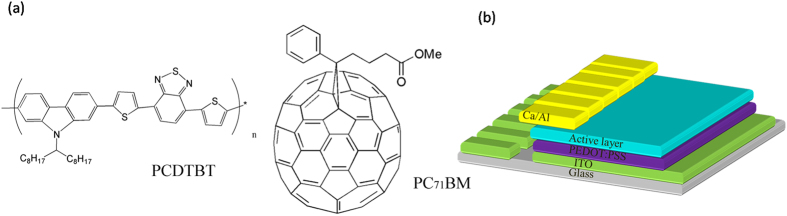
(**a**) Molecular structure of PCDTBT and PC_71_BM. (**b**) A schematic of the device structure explored.

The devices explored in this work were based on an ITO/HTL/Active layer/Ca/Al heterostructure as shown in Fig. 1(b), with each device containing 6 pixels. In this study, we have used a poly(3,4-ethylenedioxythiophene): poly(styrene sulfonic acid) (PEDOT:PSS) as the hole transport layer, as previous studies have shown that this can be used with a PCDTBT:PC_71_BM active layer to create OPVs having optimal operational stability^38^. Here, devices were built upon glass substrates with pre-patterned ITO provided by Ossila Ltd. PCDTBT was synthesized using previously reported methods^39^; and had a number molecular weight (Mn) of 15400 Da and a polydispersity of 2.21. PEDOT:PSS (HC Stark CleviosAI4083) and PC_71_BM were purchased from Ossila Ltd. The molecular structure of PCDTBT and PC_71_BM is shown in Fig. 1(a). On fabrication, devices cast from a CF, CB and CS_2_/Acetone solution had a PCE of 5.04%, 5.36% and 6.24% respectively (see device metrics in Supplementary Table S1). The enhanced efficiency of devices cast from the non-halogenated CS_2_/Acetone solvent blend is thought to result from enhanced crystallisation of the PC_71_BM^36^.

**Figure 2:**
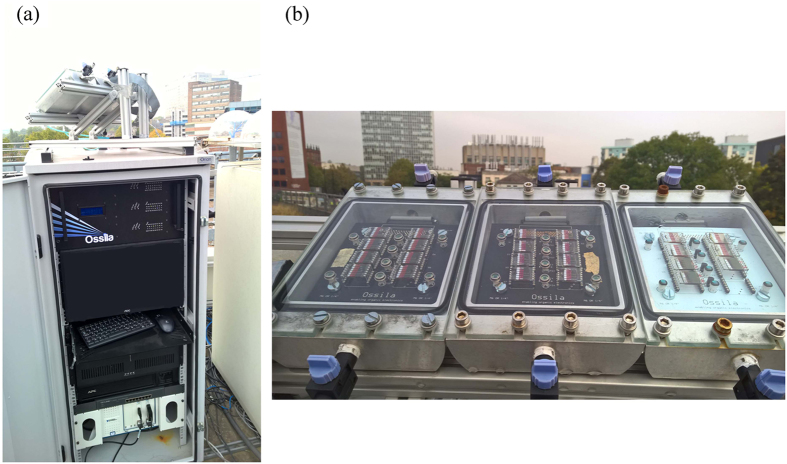
(**a**) The rooftop outdoor lifetime testing system. (**b**) The sample chambers in which devices were tested.

Before going to the real-world testing, the devices were characterized using a Newport 92251A-1000 AM 1.5 solar simulator which had been calibrated using an NREL standard silicon solar cell (Newport 91150 with PVM 387) to obtain an irradiance level of 1000 W/m^2^. Devices were then transferred to an outside laboratory for extended lifetime testing, however device metrics were also re-measured using the Newport solar simulator in laboratory approximately every two months. The outside laboratory is sited on the roof of Hicks Building at the University of Sheffield at a latitude and longitude of 53°22′N 1°29′W and is un-shaded. The testing system comprised of a hermetically sealed, aluminium sample chamber that had a toughened glass lid. During operation and testing, each chamber was filled with an overpressure of nitrogen gas offering the devices protection from the environment. All devices were mounted onto a test-board and were individually tested using a PXI-based multiplexer system controlled by a computer which determined key device metrics including power conversion efficiency (PCE), short circuit current (J_sc_), open circuit voltage (V_oc_) and fill factor (FF). For outdoor testing, a pyranometer (SPN 1 Sunshine Pyranometer from Delta-T Devices) was used to measure total irradiance over a wide range of angles of incidence and used as the primary reference for normalising the data with respect to intensity. To check for uniformity and consistency across the different substrates on the test board as well as for cross-comparison between indoor and outdoor measurements, 8 small silicon photodiodes equipped with a visible colour filter restricting sensitivity to the range of 350 to 750 nm (Vishay VBPW21R) were mounted directly on the test boards around the OPV devices under test. The multiplexer, PXI system and computer were housed in a metal cabinet as shown in Fig. 2(a). The sample chambers were mounted on the top of the electronics cabinet and faced south at an angle of 30° to the horizon as shown in Fig. 2(b).

In the measurements described below, 6 devices were characterised (two devices per casting-solvent), with all device substrates housed in the same test-chamber. The devices were tested sequentially, with measurements conducted continuously from 6 am to 9 pm every day.

Our experiments commenced on 18^th^ September 2014 (corresponding to early-autumn in England), and ended on 20^th^ September 2015, covering a period of a complete year. Clearly, the exposure conditions experienced by the OPVs are very different from those under an AM1.5 solar simulator in a lab, and thus it was necessary to relate the device performance recorded under fluctuating light levels to that recorded under standard test conditions. To do this, we have linearly normalized the J_sc_ (and thus PCE) determined at each point in time to a value expected for a (AM1.5) light exposure of 1000 W/m^2^. This normalization was performed on the basis of the light level recorded by the pyranometer. However it is well-known that the PCE measured at any one irradiance level cannot be exactly determined on the basis of this normalization process^29^. Indeed, this is confirmed by our control experiments using a laboratory-based solar simulator (see data shown in Figure S1 of Supporting Information), where we find a variation in the PCE by around 7% after linearly normalizing the PCE to different irradiance levels (between 0.25 sun and 1 sun). To further explore this, we grouped out-door device measurements into three data-sets which are dependent on the irradiance level under which they were measured (950–1050 W/m^2^, 450–550 W/m^2^ and 200–300 W/m^2^). The JV curves recorded within each light level were then adjusted using the light intensity recorded by the pyranometer, permitting the PCE and J_sc_ to be related to an irradiance level of 1000 W/m^2^. For each group of light-level measurements, we then compared the PCE, J_sc,_ V_oc_ and FF measurements made throughout the year to data recorded on the first day of the experiment. Following analysis of the data (see Supporting information Figure S2), it was found that the change in device metrics over time recorded at the different light levels were very similar and thus we combined this data into a single data-set for each device metric. While this process does not eliminate errors resulting from measurements made at different light levels, it at least permits us to follow the performance of the devices over the year (particularly during the winter months when ambient light levels are much lower).

**Figure 3:**
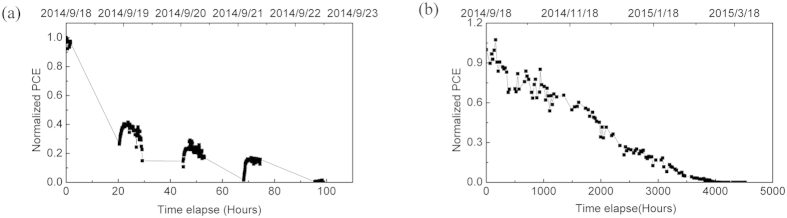
Part (**a**) shows the PCE of a typical edge pixel (1 or 6) as a function of time. Part (**b**), shows PCE for pixel next to an edge pixel (2 or 5).

**Figure 4:**
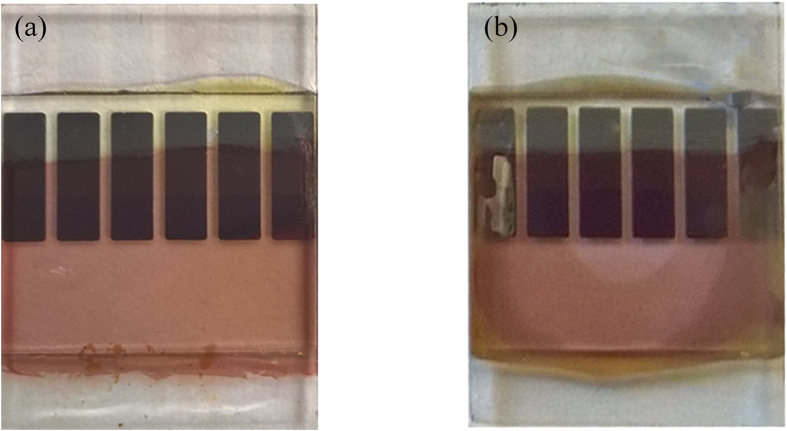
Photographs of PCDTBT:PC_71_BM based OPVs (**a**) a typical device after fabrication and (**b**) a typical device after outdoor testing for one year.

We first discuss data recorded from the pixels at the edges of the device array, i.e. pixels 1 and 6 as shown in Fig. 1(b). Our measurements showed that such edge pixels degraded quickly, with their PCE dropping to zero after 5 days measurement. Pixels 2 and 5 performed much better and survived for about 3500 hours before their efficiency dropped to zero. The reduction in PCE of a typical pixel at the edge of the substrate (positions 1 and 6) and a pixel towards the centre of the substrate (positions 2 and 5) are presented in Fig. 3. It can be seen that for pixels in positions 1, 2, 5 and 6, we determine no obvious burn-in process, with device efficiency dropping in an almost linear fashion. We attribute the rapid degradation of such pixels to the progressive edge-ingress of water and oxygen into the device. (Note that although the devices were housed in a hermetically sealed chamber that was back-filled with nitrogen, we suspect that as the devices were inserted/removed periodically for indoor testing under a solar simulator, water was repeatedly adsorbed onto the chamber walls and then slowly migrated towards the active pixels.) Indeed, our previous work indicates that the ingress from the edge of an OPV to its centre is facilitated via the presence of the hydroscopic PEDOT:PSS^40^. The importance of sealing the edges of devices has been presented by other study as well^19^. Since pixels 1 and 6 are located at the edge of the device substrate, it is unsurprising that these pixels degrade most rapidly. Indeed, after one year, that cathodes of such pixels at the edge of the substrate appear highly degraded (see Fig. 4(a,b)). It is also likely that a break-down of the encapsulation contributes to device degradation; indeed after one year, we find that the colour of the epoxy glue used to fix the cover-slip in place had turned from yellow to yellow-green. As the glass lid of the chamber had >90% optical transmission for λ > 350 nm, then exposure of the device to some fraction of the sun’s UVA spectrum is likely to result in photo-chemical changes to the epoxy that are anticipated to change its lamination/barrier properties. However, by use of a 400 nm UV filter this could presumably be minimised.

**Figure 5:**
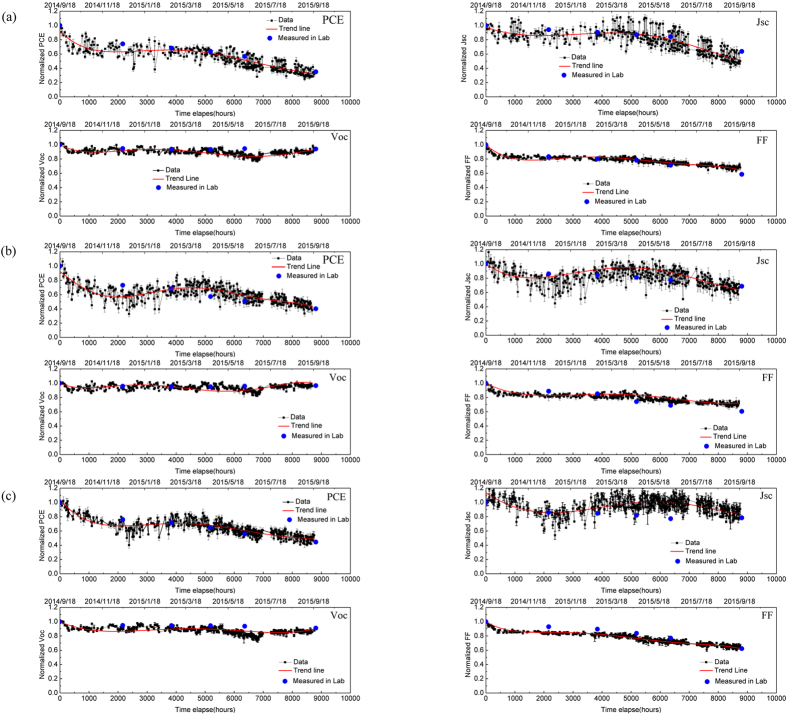
Evolution of the normalized parameters as a function of time of OPVs whose active layer was spin-cast from (**a**) CB, (**b**) CF and (**c**) CS_2_/Acetone.

The pixels at the centre of the substrate (3 and 4) are significantly more stable. We plot the PCE, J_sc_, V_oc_ and FF of such pixels in Fig. 5, with data presented from devices fabricated from the three different casting solvents. As discussed above, PCE and J_sc_ have been normalised to the ambient light-level as measured by the pyranometer. On the same plot, we include the device metrics measured in a laboratory using an AM1.5 solar simulator (solid data points). Encouragingly, we find close correspondence between device metrics recorded using both techniques, giving us confidence in the normalization process. For completeness, we also plot J-V curves recorded in the laboratory in Supporting Information.

**Figure 6:**
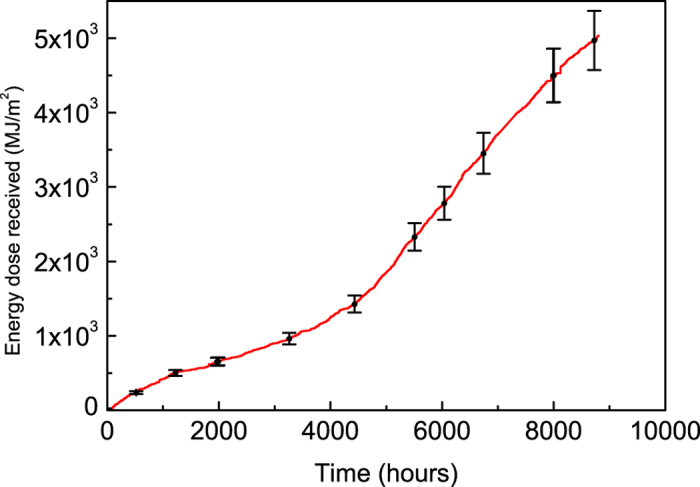
Cumulative energy dose as a function of time as received by the devices tested outdoors.

It can be seen that all the devices undergo a burn-in process during which the V_oc_ and FF degrade rapidly, with the J_sc_ undergoing a relatively smaller reduction. Such phenomena have also been observed when PCDTBT:PC_71_BM OPVs have been aged under laboratory conditions^34^. This burn-in process has been attributed to light-induced reactions in the active layer that lead to the formation of sub-band gap states. An important feature of the burn-in process is that it appears to stop after a rapid initial loss of performance, suggesting that the reacting species become depleted after sufficient exposure to light. In our previous lifetime study on PCDTBT:PC_71_BM OPVs carried out in laboratory, we determined a burn-in period of about 60 hours^38^. The burn-in period in the outdoor tests was almost 10 times longer (around 450 hours) – an effect that we ascribe the lower average light-flux experienced by the devices operating under real-world conditions (~100 W/m^2^ compared to 1000 W/m^2^). To make a more quantitative comparison, we plot the cumulative energy dose received by the devices tested outdoors as a function of time measured using the pyranometer as shown in Fig. 6. Here, the systematic uncertainty on this figure comes from a number of sources, including (i) instrumental error (2%), (ii) transmission loss through the chamber window as a result of reflection from the front and back surfaces (8%), (iii) optical absorption within the chamber window at wavelengths >3 microns (1%), and (iv) an error resulting from a difference in the location of the pyranometer and the test chamber (error at present unknown but likely small).

Using this, we estimate that the accumulative energy dose received after the first 450 hours was approximately (215 ± 20) MJ/m^2^. This corresponds to a period of 60 hours for devices exposed to a constant light intensity of 1000 W/m^2^, confirming that the length of the burn-in period is directly proportional to the incident light flux.

**Figure 7:**
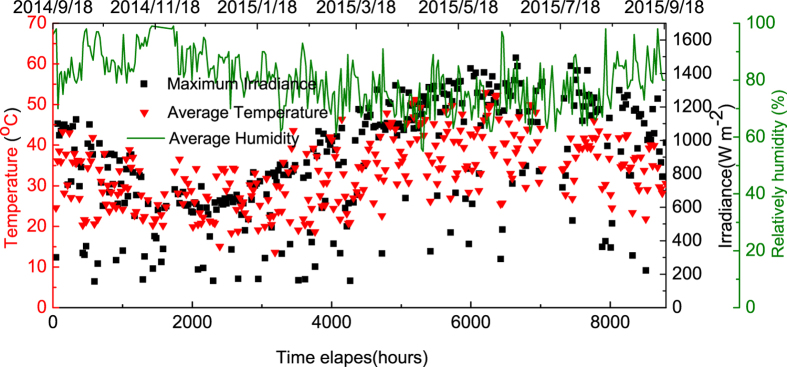
Irradiance, chamber-temperature and external-humidity recorded over the course of one year.

We recorded the average temperature within the chamber, the average external-humidity and the maximum irradiance over the course of the year, with data presented in Fig. 7. We emphasize that the temperature sensor was located inside the chamber close to the devices, and this temperature was always higher than that of the ambient air temperature outside the chamber. Figure 7 indicates that the chamber temperature follows that of the irradiance. Returning to Fig. 5, it can be seen that J_sc_ undergoes an apparent increase after 2000 hours; a process that closely correlates with a seasonal rise in the average temperature. This indicates a positive relationship between J_sc_ and operational temperature; an observation also made in other studies^33^,^34^,^41^. The mechanism that underlies this process can be assigned to temperature-dependent charge-carrier transport leading to suppressed electron-hole recombination. It can be seen from Fig. 5 that at around 5200 hours, the J_sc_ starts to degrade more rapidly. This observed reduction in J_sc_ is reflected by a concomitant reduction in PCE. In contrast however, the V_oc_ is relatively stable over the period post burn-in, with the FF reducing at a low but constant rate. At this point, changes in J_sc_ do not appear to correlate with a change in chamber temperature, and thus we ascribe this effect to an additional device degradation process. We note that this enhanced degradation coincides with the start of British summer-time, and thus we speculate that the enhanced temperature within the chamber accelerates the degradation of the encapsulation or the OPV itself via thermal-cycle stress^27^. For completeness, we plot the daily temperature recorded in the chamber over a sunny summer day and a cloudy winter day in Figure S3 in Supporting Information. We find that in summer, the temperature inside the chamber can change at a rate of 10°C/hour, reaching a maximum temperature of 75°C; a process that we believe causes thermally induced stress between the different layers in the device resulting in additional path-ways for the diffusion of oxygen or moisture into the film. Alternately, this may point to a thermal or UV-assisted break-down of the encapsulation-epoxy which then results in enhanced photo-oxidation of the active organic layer. Note that we cannot yet discount the possibility that the extended exposure to elevated temperatures results in some degree of nano-scale phase-separation within the active PCDTBT:PC_71_BM layer. More work is clearly required to understand such processes; however we believe that device stability is likely to be enhanced by improved encapsulation.

To explore whether device stability is dependent on the solvent used to cast the active layer, we tabulate the relative performance of each of the device metrics relative to its initial level in Table S3 for the two central pixels of the substrates that were cast from each solvent (equivalent to 4 pixels per solvent-type). We find that the relative changes in the FF and V_oc_ for devices cast from each type of solvent are similar. There is however some spread in the J_sc_ of devices cast from the different solvents, with the greatest reduction in J_sc_ apparently observed in devices cast from CB. We note that the statistical significance of this observation is questionable, however it may suggest that enhanced degradation occurs in devices that are cast from solvents having a higher boiling point. More measurements on a larger number of devices under accelerated aging conditions are needed to confirm such effects.

The evolution of PCE clearly results from the combined effects of J_sc_, V_oc_ and FF, and thus after one year operating under real-world conditions, we find that the PCE of devices cast from CB, CF and CS_2_/Acetone have been reduced to (30±4)%, (40±6)%, and (42±3)% of their initial level. As is conventional, we determine the device T_S_80 lifetime from the time at which the device efficiency falls to 80% of its initial value at the end of the burn-in process (here starting at 450 hours). Using this approach, we determine T_S_80 lifetimes for devices cast from CB, CS_2_/Acetone and CF to be (5200 ± 210), (5700 ± 180) and (6200 ± 270) hours respectively. Our measurements suggest therefore that devices cast from CB have the shortest T_S_80 lifetime, although more data is needed on a larger number of devices to explore the statistical significance of this observation.

In summary, PCDTBT:PC_71_BM based PSCs were fabricated from three different solvents/solvent blend and encapsulated with epoxy glue and glass slides. Device operation was then tracked during operation in an outdoor laboratory for a period of one year. We find that pixels near the edge of device substrate have reduced stability due to the ingress of water and oxygen through the device layers and through the encapsulation epoxy. Pixels close to the centre of the device substrate have enhanced stability, with T_S_80 lifetimes (post burn-in) recorded of up to 6000 hours. Our study shows that the PCDTBT:PC_71_BM based PSCs have good stability in outdoor conditions over the course of a year. Several factors may contribute to the degradation, including thermally induced stress in the device, failure of device encapsulation leading to the ingress of small amounts of water and air and photooxidation of the active organic layer. To identify the dominant degradation process, additional systematic experiments are planned. We believe that by replacing the hygroscopic PEDOT:PSS hole extraction layer with a metal oxide and utilising an inverted device architecture, further improvements in device stability are anticipated^42^. Enhanced device stability under real-world operation is also expected through the use of more sophisticated encapsulation techniques, together with the use of UV400 filtration.

## Methods

### Device fabrication

To prepare devices, ITO substrates were sequentially cleaned by ultrasonic cleaning in 10% (wt%) sodium hydroxide solution, Hellmanex solution, 2-propanol (IPA) and deionised water. The substrates were then dried using a nitrogen-gas gun and baked at 120 °C for 5 minutes to remove any adsorbed moisture. The PEDOT:PSS was then spin cast onto the substrates at a spin-speed of 5000 rpm for 30 seconds to create a film having a thickness of ~30 nm. The substrates were again annealed at 120 °C for 5 minutes before being transferred to a nitrogen-filled glove box. To prepare the active layer, PCDTBT and PC_71_BM were mixed at a ratio of 1:4 by weight and dissolved in a solvent (either CB, CF or CS_2_/Acetone) at a total concentration of 20, 18 and 18 mg/ml, respectively. Here, spin-speed was adjusted to form an active layer having a thickness of 70 nm. A cathode (5 nm calcium and 100 nm aluminium) was then thermally evaporated onto the active-layer surface through a shadow mask using a thermal evaporator under base pressure of 2 × 10^−6 ^mBar creating a device having an active area of 4.8 mm^2^. Finally, OPVs were encapsulated using a glass cover-slip and a UV-curable epoxy.

### Device characterisation in lab-condition

In the laboratory, the devices were characterized using a Newport 92251A-1000 AM 1.5 solar simulator which had been calibrated using an NREL standard silicon solar cell to obtain an irradiance level of 1000 W/m^2^. An aperture mask was used to limit the light-exposed area of the device to 4.5 mm^2^. The temperature of the laboratory during the tests was approximately 25 °C.

## Additional Information

**How to cite this article**: Zhang, Y. *et al*. PCDTBT based solar cells: one year of operation under real-world conditions. *Sci. Rep.* **6**, 21632; doi: 10.1038/srep21632 (2016).

## Supplementary Material

Supplementary Information
