# Supplementary material for: PCDTBT based solar cells: one year of operation under real-world conditions
Source: Sci Rep. 2016 Feb 9;6:21632. doi: 10.1038/srep21632 (PMC4746663; doi:10.1038/srep21632)
Supplement: Supplementary Information [file srep21632-s1.pdf]

Supplementary Information

# PCDTBT based solar cells: one year of operation under real-world conditions

Yiwei Zhang[1], Edward Bovill[1], James Kingsley[2], Alastair R. Buckley[1], Hunan Yi[3], Ahmed Iraqi[3], Tao Wang[4*], David G. Lidzey[1*]

[1]Department of Physics and Astronomy, University of Sheffield, Sheffield, S3 7RH, UK

[2]Ossila Ltd, Kroto Innovation Centre, Broad Lane, Sheffield, S3 7HQ, UK

[3]Department of Chemistry, University of Sheffield, Sheffield S3 7HF, UK

[4]School of Materials Science and Engineering, Wuhan University of Technology, Wuhan, 430070, China

Corresponding: twang@whut.edu.cn, d.g.lidzey@sheffield.ac.uk

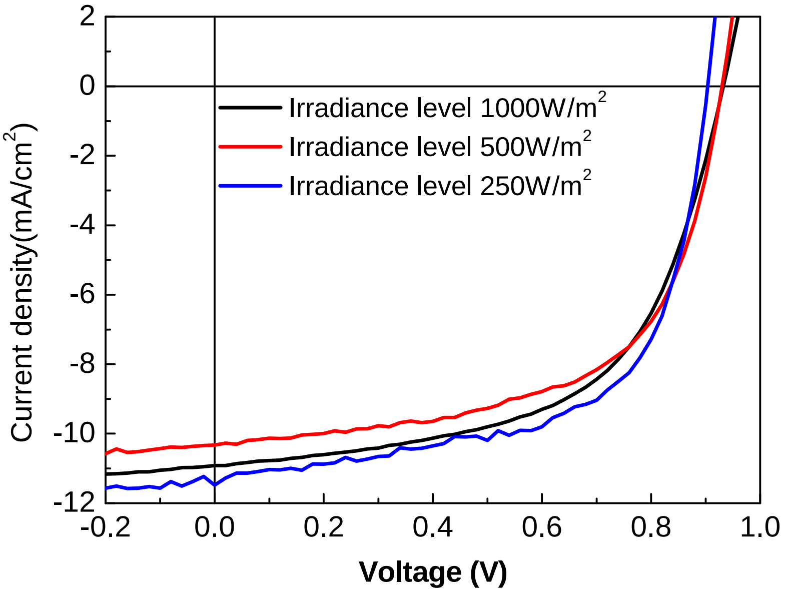


Figure S1: The J-V curves of devices measured under different irradiance levels in laboratory and then normalized to an irradiance intensity of 1000W/$m^2$.

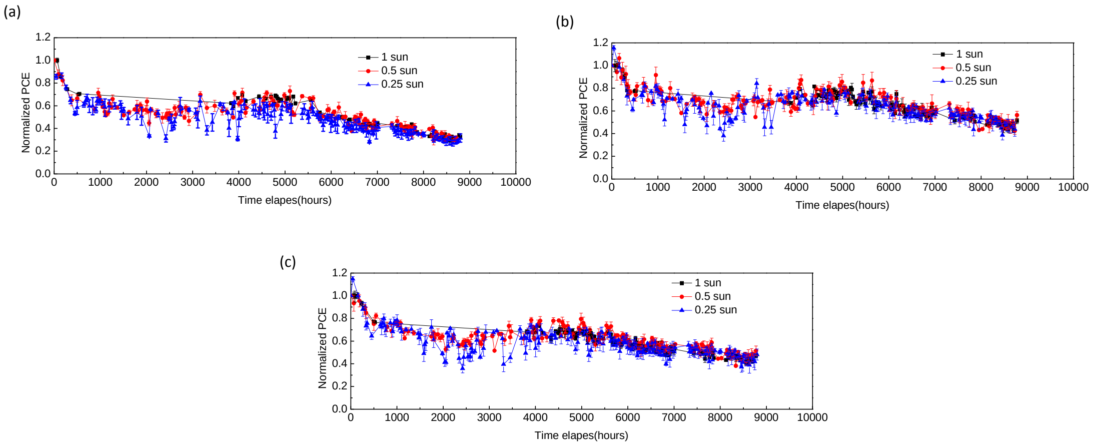


Figure S2 Evolution of PCE normalized to different irradiance level as a function of time of OPVs whose active layer was spin-cast from (a) CB, (b) CF and (c) $CS_2$/Acetone

Table S1: Metrics of a device measured under different irradiance levels in laboratory and then normalized to an irradiance intensity of 1000W/m$^2$.

| Irradiance level (W/m$^2$) | 1000 | 500 | 250 |
|---|---|---|---|
| PCE (%) | 5.90 | 5.73 | 6.33 |
| $J_{sc}$ (mA/cm$^2$) | -10.91 | -10.32 | -11.49 |
| $V_{oc}$ (V) | 0.93 | 0.93 | 0.90 |
| FF (%) | 58.00 | 59.64 | 60.95 |

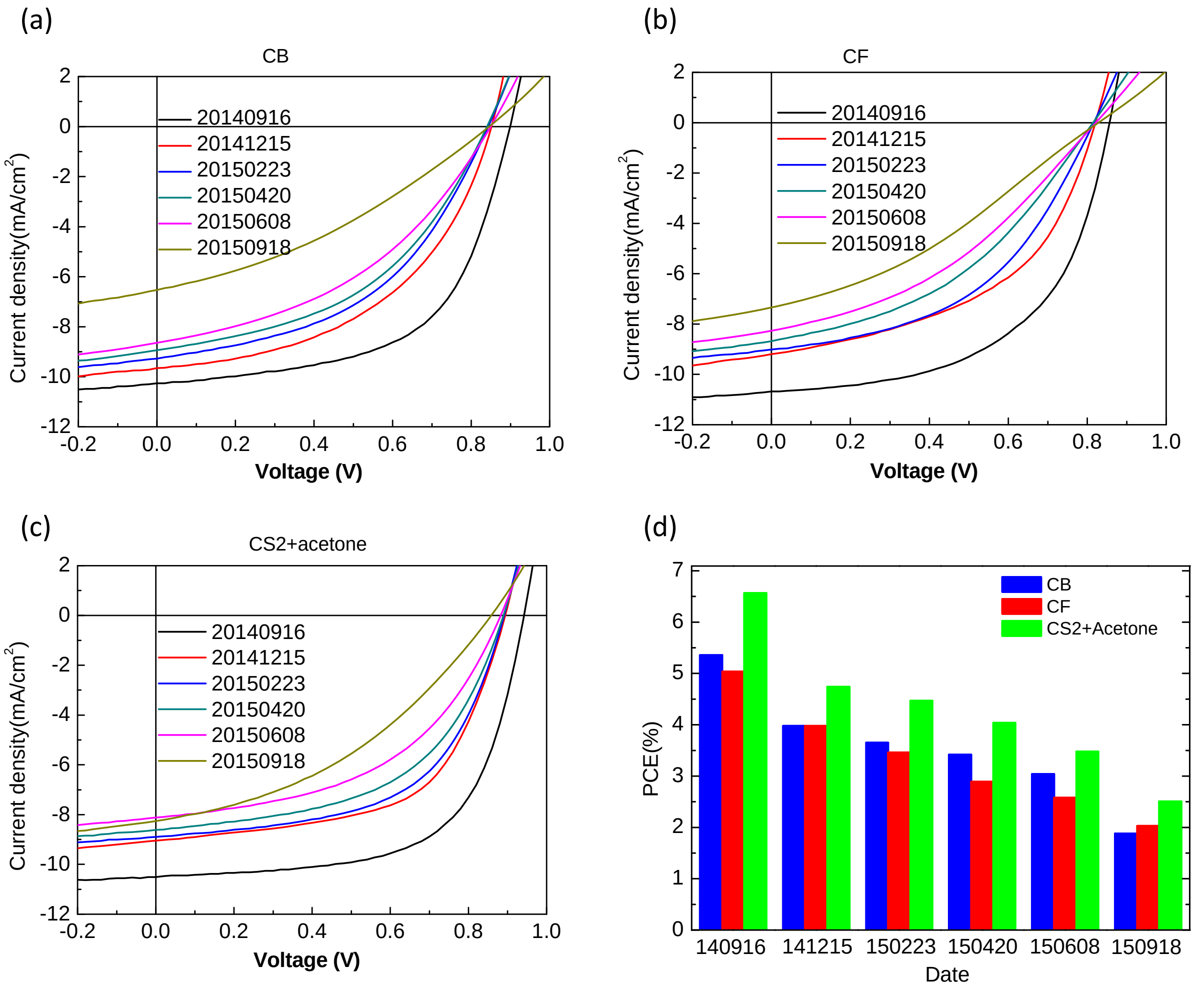


Figure S3: J-V curves of devices cast from (a) CB, (b) CF, (c) $CS_2$/Acetone measured in laboratory and (d) a summary of the efficiency recorded on different dates.

Table S2: Device metrics measured in the laboratory on different testing dates.

| Solvent | Metrics | 20140916 | 20141215 | 20150223 | 20150420 | 20150608 | 20150918 |
|---|---|---|---|---|---|---|---|
| CB | PCE (%) | 5.36 | 3.98 | 3.65 | 3.41 | 3.04 | 1.88 |
| | $J_{sc}$ (mA/cm$^2$) | -10.25 | -9.65 | -9.27 | -8.94 | -8.65 | -6.53 |
| | $V_{oc}$ (V) | 0.90 | 0.85 | 0.84 | 0.84 | 0.85 | 0.85 |
| | FF (%) | 58.14 | 45.48 | 46.74 | 45.51 | 41.42 | 34.05 |
| CF | PCE (%) | 5.04 | 3.69 | 3.46 | 2.89 | 2.58 | 2.03 |
| | $J_{sc}$ (mA/cm$^2$) | -10.68 | -9.20 | -9.01 | -8.68 | -8.26 | -7.34 |
| | $V_{oc}$ (V) | 0.86 | 0.82 | 0.82 | 0.81 | 0.82 | 0.83 |
| | FF (%) | 55.16 | 48.96 | 47.00 | 40.92 | 38.06 | 33.41 |
| $CS_2$/Acetone | PCE (%) | 6.24 | 4.74 | 4.47 | 4.04 | 3.48 | 2.78 |
| | $J_{sc}$ (mA/cm$^2$) | -10.51 | -9.04 | -8.99 | -8.61 | -8.12 | -8.25 |
| | $V_{oc}$ (V) | 0.94 | 0.89 | 0.89 | 0.89 | 0.89 | 0.86 |
| | FF (%) | 63.00 | 58.59 | 56.42 | 52.74 | 48.55 | 39.24 |

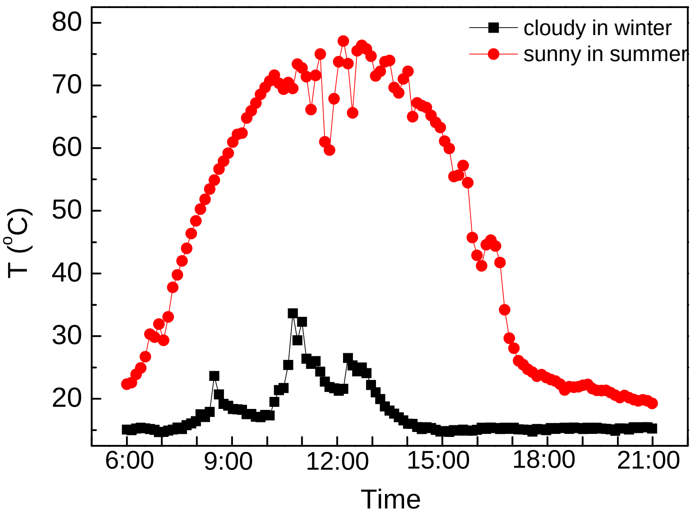


Figure S4: Comparison of measured temperature variation in the device testing chamber on a cloudy day in winter and a sunny day in summer.

Table S3 The statistics of degradation after outdoor testing for one year for the two central pixels (3 and 4) from two different substrates. Data is tabulated for devices cast from the three different solvents.

| Solvent | CB | | CF | | $CS_2$/acetone | |
|---|---|---|---|---|---|---|
| Relative PCE after 1 year ($PCE_{1y}/PCE_0$)% | Pixel 1 | 29% | Pixel 1 | 31% | Pixel 1 | 43% |
| | Pixel 2 | 31% | Pixel 2 | 42% | Pixel 2 | 45% |
| | Pixel 3 | 35% | Pixel 3 | 40% | Pixel 3 | 42% |
| | Pixel 4 | 25% | Pixel 4 | 45% | Pixel 4 | 38% |
| | **average** | **30%±4%** | **average** | **40%±6%** | **average** | **42%±3%** |
| Relative $J_{sc}$ after one year ($Jsc_{1y}/Jsc_0$)% | Pixel 1 | 61% | Pixel 1 | 53% | Pixel 1 | 74% |
| | Pixel 2 | 59% | Pixel 2 | 67% | Pixel 2 | 79% |
| | Pixel 3 | 64% | Pixel 3 | 69% | Pixel 3 | 76% |
| | Pixel 4 | 47% | Pixel 4 | 72% | Pixel 4 | 63% |
| | **average** | **58%±7%** | **average** | **65%±8%** | **average** | **73%±7%** |
| Relative $V_{oc}$ after 1 year ($Voc_{1y}/Voc_0$)% | Pixel 1 | 93% | Pixel 1 | 96% | Pixel 1 | 91% |
| | Pixel 2 | 93% | Pixel 2 | 97% | Pixel 2 | 91% |
| | Pixel 3 | 94% | Pixel 3 | 97% | Pixel 3 | 93% |
| | Pixel 4 | 93% | Pixel 4 | 96% | Pixel 4 | 94% |
| | **average** | **93%±0%** | **average** | **96%±1%** | **average** | **92%±2%** |
| Relative FF after one year ($FF_{1y}/FF_0$)% | Pixel 1 | 51% | Pixel 1 | 62% | Pixel 1 | 64% |
| | Pixel 2 | 57% | Pixel 2 | 64% | Pixel 2 | 62% |
| | Pixel 3 | 57% | Pixel 3 | 61% | Pixel 3 | 59% |
| | Pixel 4 | 58% | Pixel 4 | 65% | Pixel 4 | 65% |
| | **average** | **56%±3%** | **average** | **63%±2%** | **average** | **62%±3%** |
